# Supplementary material for: High performance sorting of motor unit action potentials with EMUsort
Source: bioRxiv. 2026 Jan 7:2026.01.06.697952. Preprint. [Version 1] doi: 10.64898/2026.01.06.697952 (PMC12803147; doi:10.64898/2026.01.06.697952)
Supplement: Supplement 1 [file NIHPP2026.01.06.697952v1-supplement-1.pdf]

bioRxiv preprint doi: https://doi.org/10.64898/2026.01.06.697952; this version posted January 7, 2026. The copyright holder for this preprint (which was not certified by peer review) is the author/funder, who has granted bioRxiv a license to display the preprint in perpetuity. It is made available under aCC-BY 4.0 International license.

Additional Files

Supplementary Tables

Table 1 - table supplement 1: Descriptive statistics for distributions of cross-unit mean accuracy scores for each sort run with the rat simulated dataset, using the definition of accuracy as computed in (Pachitariu et al., 2024). Values in brackets were computed using only MUAPs that overlapped within half the template width of another MUAP.

		Descriptive Statistics for Distributions of Cross-Unit Mean Accuracy Scores		
**Sorter**	**Run Count**	**Median (%)** all spikes [overlaps]	**Mean (%)** all spikes [overlaps]	**Standard Deviation (%)** all spikes [overlaps]
EMUsort	25	94.8 [84.1]	91.9 [79.8]	6.2 [8.9]
Kilosort4	25	81.2 [51.1]	76.4 [48.5]	12.3 [13.4]
MUedit	4	41.3 [16.3]	45.0 [19.3]	14.2 [15.7]

Table 1 - table supplement 2: Descriptive statistics for distributions of cross-unit mean error rates for each sort run with the rat simulated dataset. This is computed by taking the accuracy scores from Table 1 - table supplement 1 for each sort run and subtracting them into 1. Values in brackets were computed using only MUAPs that overlapped within half the template width of another MUAP.

		Descriptive Statistics for Distributions of Cross-Unit Mean Error Rates		
**Sorter**	**Run Count**	**Median (%)** all spikes [overlaps]	**Mean (%)** all spikes [overlaps]	**Standard Deviation (%)** all spikes [overlaps]
EMUsort	25	5.2 [15.9]	8.1 [20.2]	6.2 [8.9]
Kilosort4	25	18.8 [48.9]	23.6 [51.5]	12.3 [13.4]
MUedit	4	58.7 [83.7]	55.0 [80.7]	14.2 [15.7]

Table 1 - table supplement 3: Per-unit performance statistics from the best EMUsort run, computed with all spikes from the rat simulated dataset.

Unit	True Count	Sorter Count	Precision	Recall	Accuracy
1	21704	20933	99.8%	96.2%	96.0%
2	16484	15064	99.3%	90.7%	90.1%
3	12529	12126	100.0%	96.8%	96.8%
4	11041	10929	99.9%	98.9%	98.8%
5	8438	8401	98.6%	98.2%	96.8%
6	6052	5999	100.0%	99.1%	99.1%
7	4190	4188	99.3%	99.2%	98.5%
8	4143	4151	99.8%	100.0%	99.8%
9	3178	3133	99.2%	97.8%	97.0%
10	1330	1333	99.4%	99.6%	99.0%

bioRxiv preprint doi: https://doi.org/10.64898/2026.01.06.697952; this version posted January 7, 2026. The copyright holder for this preprint (which was not certified by peer review) is the author/funder, who has granted bioRxiv a license to display the preprint in perpetuity. It is made available under aCC-BY 4.0 International license.

		**Median**	99.6%	98.5%	97.7%
		**Mean**	99.5%	97.7%	97.2%
		**Standard Deviation**	0.5%	2.7%	2.8%

Table 1 - table supplement 4: Per-unit performance statistics from the best sort run with EMUsort, computed only with overlapping spikes from the rat simulated dataset.

Unit	True Count	Sorter Count	Precision	Recall	Accuracy
1	8465	7150	96.7%	81.7%	79.5%
2	7651	6195	95.7%	77.5%	74.8%
3	6688	5941	97.9%	87.0%	85.4%
4	6194	5907	97.5%	93.0%	90.8%
5	5235	5118	95.6%	93.4%	89.5%
6	4092	3958	98.4%	95.2%	93.7%
7	3379	3382	96.1%	96.2%	92.6%
8	3351	3362	96.6%	96.9%	93.7%
9	2440	2341	95.0%	91.1%	86.9%
10	1114	1108	97.8%	97.3%	95.3%
		**Median**	96.7%	93.2%	90.2%
		**Mean**	96.7%	90.9%	88.2%
		**Standard Deviation**	1.1%	6.8%	6.7%

Table 1 - table supplement 5: Per-unit performance statistics from the best KS4 run, computed with all spikes from the rat simulated dataset.

Unit	True Count	Sorter Count	Precision	Recall	Accuracy
1	21704	21189	99.5%	97.2%	96.7%
2	16484	15205	99.9%	92.2%	92.1%
3	12529	12273	100.0%	97.9%	97.9%
4	11041	10956	98.8%	98.0%	96.8%
5	8438	6837	90.7%	73.5%	68.4%
6	6052	5845	98.3%	95.0%	93.4%
7	4190	4117	97.3%	95.6%	93.1%
8	4143	4125	98.4%	98.0%	96.5%
9	3178	3056	96.0%	92.4%	89.0%
10	1330	1314	99.9%	98.7%	98.6%
		**Median**	98.6%	96.4%	95.0%

bioRxiv preprint doi: https://doi.org/10.64898/2026.01.06.697952; this version posted January 7, 2026. The copyright holder for this preprint (which was not certified by peer review) is the author/funder, who has granted bioRxiv a license to display the preprint in perpetuity. It is made available under aCC-BY 4.0 International license.

		**Mean**	97.9%	93.8%	92.3%
		**Standard Deviation**	2.8%	7.5%	8.9%

Table 1 - table supplement 6: Per-unit performance statistics from the best sort run with KS4, computed only with overlapping spikes from the rat simulated dataset.

Unit	True Count	Sorter Count	Precision	Recall	Accuracy
1	8465	7352	87.4%	75.9%	68.4%
2	7651	6212	92.0%	74.7%	70.2%
3	6688	6114	92.1%	84.2%	78.5%
4	6194	5816	90.4%	84.8%	77.8%
5	5235	3761	75.7%	54.4%	46.3%
6	4092	3691	89.5%	80.8%	73.8%
7	3379	3220	93.6%	89.2%	84.1%
8	3351	3243	93.6%	90.6%	85.4%
9	2440	2260	85.8%	79.5%	70.3%
10	1114	1068	92.6%	88.8%	82.9%
		**Median**	91.2%	82.5%	75.8%
		**Mean**	89.3%	80.3%	73.8%
		**Standard Deviation**	5.4%	10.6%	11.4%

Table 1 - table supplement 7: Per-unit performance statistics from the best MUedit run, computed with all spikes from the rat simulated dataset.

Unit	True Count	Sorter Count	Precision	Recall	Accuracy
1	21704	20942	98.6%	95.2%	94.0%
2	16484	15588	98.0%	92.7%	91.0%
3	12529	12431	100.0%	99.2%	99.2%
4	11041	10802	79.1%	77.3%	64.2%
5	8438	9645	64.0%	73.1%	51.8%
6	6052	5831	98.6%	95.0%	93.8%
7	4190	4145	91.9%	90.9%	84.2%
8	4143	4033	100.0%	97.3%	97.3%
9	3178	0	0.0%	0.0%	0.0%
10	1330	1228	100.0%	92.3%	92.3%
		**Median**	98.3%	92.5%	91.7%
		**Mean**	83.0%	81.3%	76.8%

bioRxiv preprint doi: https://doi.org/10.64898/2026.01.06.697952; this version posted January 7, 2026. The copyright holder for this preprint (which was not certified by peer review) is the author/funder, who has granted bioRxiv a license to display the preprint in perpetuity. It is made available under aCC-BY 4.0 International license.

		**Standard Deviation**	31.5%	29.8%	31.1%

Table 1 - table supplement 8: Per-unit performance statistics from the best sort run with MUedit, computed only with overlapping spikes from the rat simulated dataset.

Unit	True Count	Sorter Count	Precision	Recall	Accuracy
1	8465	7277	85.1%	73.2%	64.9%
2	7651	6787	85.8%	76.1%	67.6%
3	6688	6645	86.3%	85.7%	75.4%
4	6194	6371	53.7%	55.2%	37.4%
5	5235	6788	47.3%	61.4%	36.5%
6	4092	3787	82.0%	75.9%	65.0%
7	3379	3317	83.6%	82.1%	70.7%
8	3351	3755	83.7%	93.8%	79.3%
9	2440	0	0.0%	0.0%	0.0%
10	1114	964	94.4%	81.7%	77.9%
		**Median**	83.7%	76.0%	66.3%
		**Mean**	70.2%	68.5%	57.5%
		**Standard Deviation**	28.9%	26.6%	25.3%

Table 2 - table supplement 1: Descriptive statistics for distributions of cross-unit mean accuracy scores for each sort run with the monkey simulated dataset, using the definition of accuracy as computed in (Pachitariu et al., 2024). Values in brackets were computed using only MUAPs that overlapped within half the template width of another MUAP.

		**Descriptive Statistics for Distributions of Cross-Unit Mean Accuracy Scores**		
**Sorter**	**Run Count**	**Median (%)** all spikes [overlaps]	**Mean (%)** all spikes [overlaps]	**Standard Deviation (%)** all spikes [overlaps]
EMUsort	25	98.1 [83.0]	97.1 [76.8]	2.7 [12.8]
Kilosort4	25	95.9 [66.1]	95.6 [67.7]	2.4 [7.1]
MUedit	4	93.4 [57.6]	90.1 [57.1]	7.5 [14.6]

Table 2 - table supplement 2: Descriptive statistics for distributions of cross-unit mean error rates for each sort run with the monkey simulated dataset. This is computed by taking the accuracy scores from each sort run and subtracting them into 1. Values in brackets were computed using only MUAPs that overlapped within half the template width of another MUAP.

		**Descriptive Statistics for Distributions of Cross-Unit Mean Error Rates**		
**Sorter**	**Run Count**	**Median (%)** all spikes [overlaps]	**Mean (%)** all spikes [overlaps]	**Standard Deviation (%)** all spikes [overlaps]
EMUsort	25	1.9 [17.0]	2.9 [23.2]	2.7 [12.8]
Kilosort4	25	4.1 [33.9]	4.4 [32.3]	2.4 [7.1]
MUedit	4	6.6 [42.4]	9.9 [42.9]	7.5 [14.6]

bioRxiv preprint doi: https://doi.org/10.64898/2026.01.06.697952; this version posted January 7, 2026. The copyright holder for this preprint (which was not certified by peer review) is the author/funder, who has granted bioRxiv a license to display the preprint in perpetuity. It is made available under aCC-BY 4.0 International license.

Table 2 - table supplement 3: Per-unit performance statistics from the best EMUsort run, computed with all spikes from the monkey simulated dataset.

Unit	True Count	Sorter Count	Precision	Recall	Accuracy
1	12033	12038	99.9%	99.9%	99.8%
2	6426	6421	99.9%	99.8%	99.7%
3	3151	3151	100.0%	100.0%	100.0%
4	1960	1960	100.0%	100.0%	100.0%
5	645	645	100.0%	100.0%	100.0%
		**Median**	100.0%	100.0%	100.0%
		**Mean**	100.0%	99.9%	99.9%
		**Standard Deviation**	0.1%	0.1%	0.1%

Table 2 - table supplement 4: Per-unit performance statistics from the best sort run with EMUsort, computed only with overlapping spikes from the monkey simulated dataset.

Unit	True Count	Sorter Count	Precision	Recall	Accuracy
1	1736	1754	93.3%	94.2%	88.2%
2	1552	1559	92.7%	93.1%	86.7%
3	1085	1080	96.2%	95.8%	92.3%
4	780	774	97.2%	96.4%	93.8%
5	290	295	96.6%	98.3%	95.0%
		**Median**	96.2%	95.8%	92.3%
		**Mean**	95.2%	95.6%	91.2%
		**Standard Deviation**	2.1%	2.0%	3.6%

Table 2 - table supplement 5: Per-unit performance statistics from the best KS4 run, computed with all spikes from the monkey simulated dataset.

Unit	True Count	Sorter Count	Precision	Recall	Accuracy
1	12033	12043	98.8%	98.9%	97.8%
2	6426	6346	99.4%	98.2%	97.6%
3	3151	3156	99.6%	99.8%	99.4%
4	1960	1959	100.0%	99.9%	99.9%
5	645	644	99.8%	99.7%	99.5%
		**Median**	99.6%	99.7%	99.4%
		**Mean**	99.5%	99.3%	98.9%

bioRxiv preprint doi: https://doi.org/10.64898/2026.01.06.697952; this version posted January 7, 2026. The copyright holder for this preprint (which was not certified by peer review) is the author/funder, who has granted bioRxiv a license to display the preprint in perpetuity. It is made available under aCC-BY 4.0 International license.

		**Standard Deviation**	0.5%	0.7%	1.1%

Table 2 - table supplement 6: Per-unit performance statistics from the best sort run with KS4, computed only with overlapping spikes from the monkey simulated dataset.

**Unit**	**True Count**	**Sorter Count**	**Precision**	**Recall**	**Accuracy**
1	1736	1950	82.8%	93.0%	78.0%
2	1552	1462	92.1%	86.8%	80.8%
3	1085	1100	90.5%	91.8%	83.8%
4	780	740	91.8%	87.1%	80.7%
5	290	281	94.7%	91.7%	87.2%
		**Median**	91.8%	91.7%	80.8%
		**Mean**	90.4%	90.1%	82.1%
		**Standard Deviation**	4.5%	2.9%	3.5%

Table 2 - table supplement 7: Per-unit performance statistics from the best MUedit run, computed with all spikes from the monkey simulated dataset.

**Unit**	**True Count**	**Sorter Count**	**Precision**	**Recall**	**Accuracy**
1	12033	11144	100.0%	92.6%	92.6%
2	6426	6657	94.8%	98.2%	93.2%
3	3151	2991	100.0%	94.9%	94.9%
4	1960	1893	100.0%	96.6%	96.6%
5	645	620	100.0%	96.1%	96.1%
		**Median**	100.0%	96.1%	94.9%
		**Mean**	99.0%	95.7%	94.7%
		**Standard Deviation**	2.3%	2.1%	1.8%

Table 2 - table supplement 8: Per-unit performance statistics from the best sort run with MUedit, computed only with overlapping spikes from the monkey simulated dataset.

**Unit**	**True Count**	**Sorter Count**	**Precision**	**Recall**	**Accuracy**
1	1736	1555	97.6%	87.4%	85.6%
2	1552	1747	77.8%	87.6%	70.1%
3	1085	981	98.1%	88.7%	87.1%
4	780	902	77.7%	89.9%	71.5%
5	290	299	80.6%	83.1%	69.3%
		**Median**	80.6%	87.6%	71.5%

bioRxiv preprint doi: https://doi.org/10.64898/2026.01.06.697952; this version posted January 7, 2026. The copyright holder for this preprint (which was not certified by peer review) is the author/funder, who has granted bioRxiv a license to display the preprint in perpetuity. It is made available under aCC-BY 4.0 International license.

		**Mean**	86.4%	87.3%	76.7%
		**Standard Deviation**	10.5%	2.6%	8.9%

Supplementary Figures

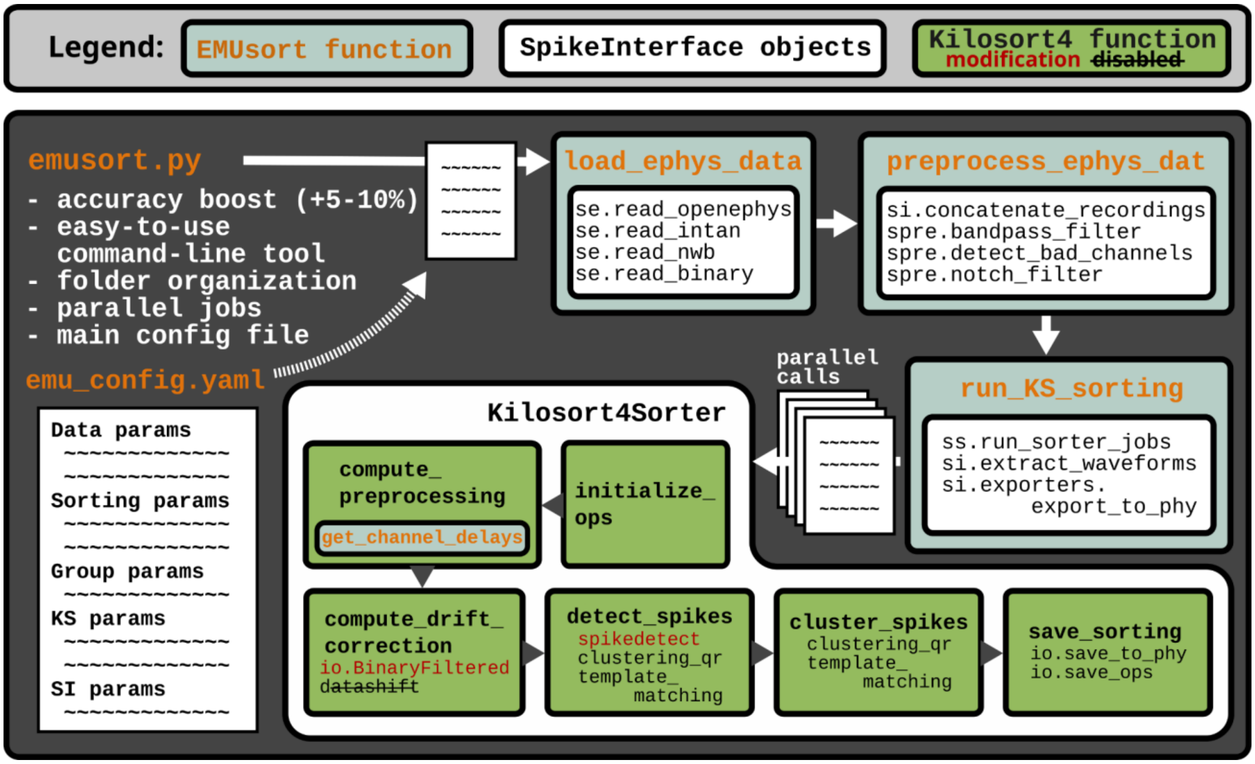

Supplementary Figure 1. EMUsort leverages open-source libraries with a modular organization. The legend at the top indicates the color coding and symbolic meanings of the various boxes and fonts. The arrows indicate the order and hierarchy of function calls. The main script for the command line interface, "emusort.py", utilizes a configuration file which stores all settings defining the input dataset properties, sorting parameters, settings defining separate groups of channels to use, and finally KS4 and SpikeInterface parameters. SpikeInterface methods are leveraged for loading, preprocessing, and starting KS4 processes in series or as parallel jobs with different settings. The modifications to KS4 source code, indicated by the legend, were implemented to remove channel delays and improve the initialization of templates.

bioRxiv preprint doi: https://doi.org/10.64898/2026.01.06.697952; this version posted January 7, 2026. The copyright holder for this preprint (which was not certified by peer review) is the author/funder, who has granted bioRxiv a license to display the preprint in perpetuity. It is made available under aCC-BY 4.0 International license.

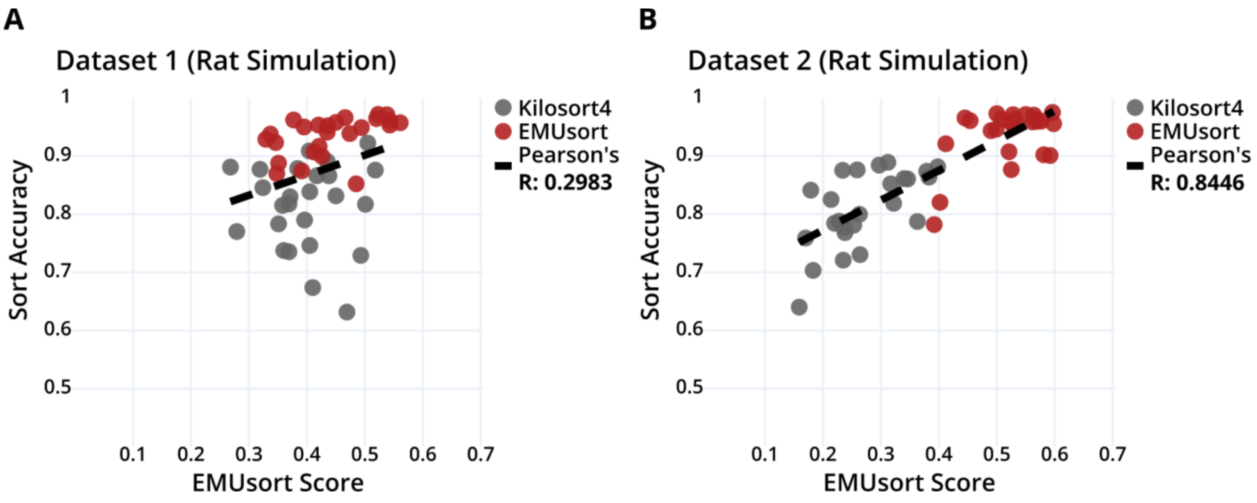

Supplementary Figure 2. The composite scores produced by EMUsort for 2 of our simulated rat datasets can serve as a rough estimate of the computed ground truth accuracies (averaged across all units for each sort) with a weak positive correlation for Dataset 1 and a strong positive correlation for Dataset 2. Dataset 1 was used for the main results generated for Figure 6. Pearson's R value is shown in the legend of each graph.

bioRxiv preprint doi: https://doi.org/10.64898/2026.01.06.697952; this version posted January 7, 2026. The copyright holder for this preprint (which was not certified by peer review) is the author/funder, who has granted bioRxiv a license to display the preprint in perpetuity. It is made available under aCC-BY 4.0 International license.

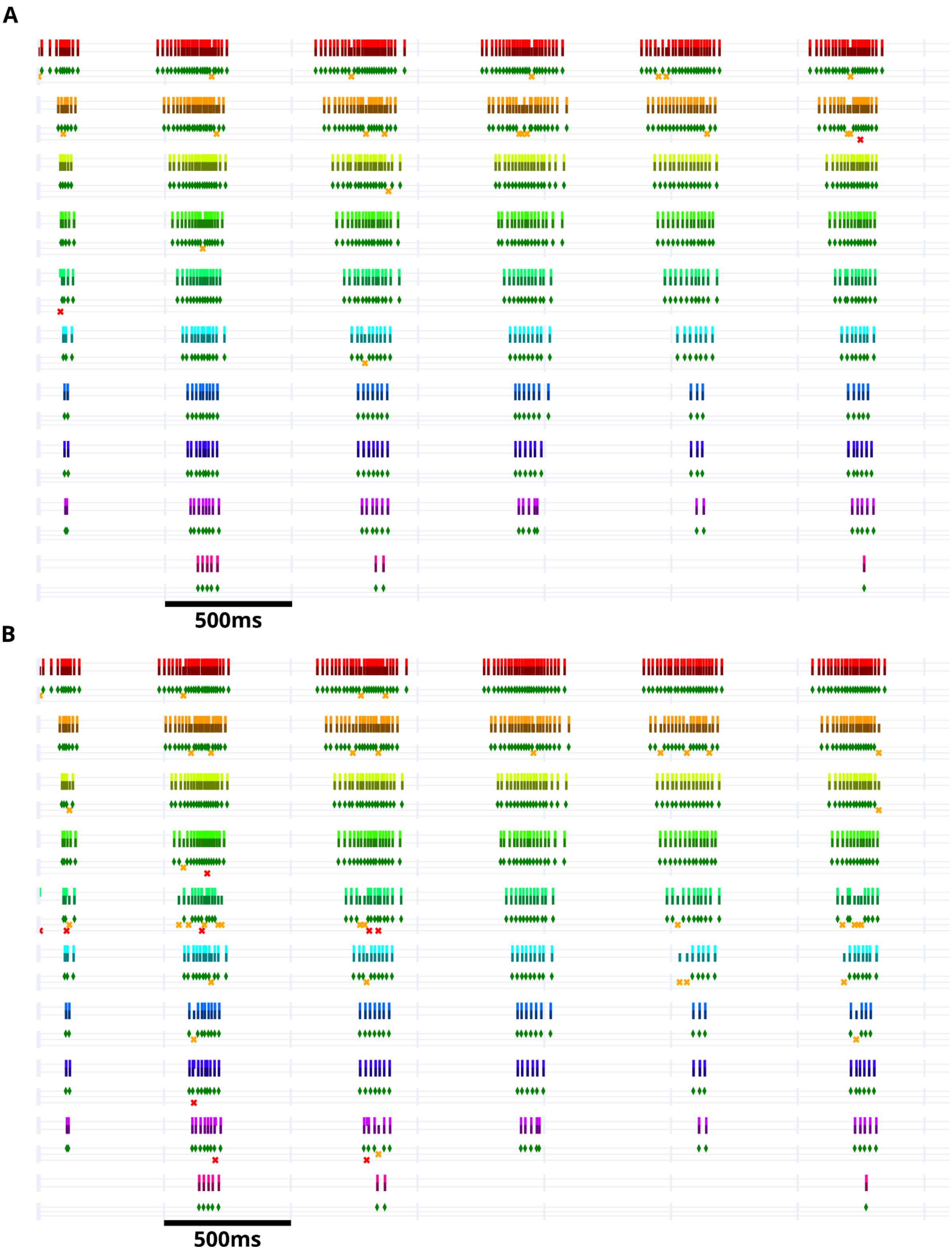

Supplementary Figure 3. Example time stretch in the rat simulated dataset showing the ground truth versus identified spike times. Each row shows spike times with uniquely colored raster ticks for each motor unit. Darker ticks for each color show the ground truth spike times. Green diamonds mark correct times within +/- 1 ms. Yellow X's mark false negatives. Red X's mark false positives. (a) Event plot showing EMUsort evaluations. (b) Event plot showing KS4 evaluations.
